# Molluscicidal and antioxidant activities of silver nanoparticles on the multi-species of snail intermediate hosts of schistosomiasis

**DOI:** 10.1371/journal.pntd.0010667

**Published:** 2022-10-10

**Authors:** Khaled M. Zayed, Yun-Hai Guo, Shan Lv, Yi Zhang, Xiao-Nong Zhou

**Affiliations:** 1 Medical Malacology Department, Theodor Bilharz Research Institute, Giza, Egypt; 2 National Institute of Parasitic Diseases, Chinese Center for Diseases Control and Prevention, Shanghai, People’s Republic of China; 3 NHC Key Laboratory of Parasite and Vector Biology; WHO Collaborating Centre for Tropical Diseases; National Center for International Research on Tropical Diseases, Ministry of Science and Technology, Shanghai, People’s Republic of China; 4 School of Global Health, Chinese Center for Tropical Diseases Research, Shanghai Jiao Tong University School of Medicine, Shanghai, People’s Republic of China; Texas Biomedical Research institute, UNITED STATES

## Abstract

**Background:**

Schistosomiasis, also known as bilharzia, is the second important parasitic disease after malaria. The present study aimed to evaluate the molluscicidal effects of silver nanoparticles on *Biomphalaria alexandrina*, *B*. *glabrata*, *Oncomelania hupensis*, snail intermediate hosts of intestinal schistosomes (i.e. *Schistosoma mansoni* and *S*. *japonicum*), along with the changes their antioxidant enzymes.

**Methods:**

Silver (Ag) nano powder (Ag-NPs) was selected to test the molluscicidal effects on three species of freshwater snails. Exposure to Ag-NPs induced snail mortality and the LC_50_ and LC_90_ values of Ag-NPs for each snail species were calculated by probit analysis. Control snails were maintained under the same experimental conditions in dechlorinated water. Snail hemolymph was collected to measure the levels of antioxidant enzymes, such as total antioxidants capacity (TCA), glutathione (GSH), catalase (CAT) and nitric oxide (NO). In addition, the non-target organism, *Daphnia magna*, was exposed to a series of Ag-NPs concentration, similar to the group of experimental snails, in order to evaluate the LC_50_ and LC_90_ and compare these values to those obtained for the targeted snails.

**Results:**

The results indicated that Ag-NPs had a molluscicidal effect on tested snails with the variation in lethal concentration. The LC_50_ values of Ag-NPs for *B*. *alexandrina* snails exposed for 24, 48, 72 hrs and 7 days were 7.91, 5.69, 3.83 and 1.91 parts per million (ppm), respectively. The LC_50_ values for *B*. *glabrata* snails exposed for 24, 48, 72 hrs and 7 days were 16.55, 10.44, 6.91 and 4.13 ppm, respectively, while the LC_50_ values for *O*. *hupensis* snails exposed for 24, 48, 72 hrs and 7 days were 46.5, 29.85, 24.49 and 9.62 ppm, respectively. Moreover, there is no mortality detected on *D*. *magna* when exposed to more than double and half concentration (50 ppm) of Ag-NPs during a continuous period of 3 hrs, whereas the LC_90_ value for *B*. *alexandrina* snails was 18 ppm. The molluscicidal effect of the synthesized Ag-NPs seems to be linked to a potential reduction of the antioxidant activity in the snail’s hemolymph.

**Conclusions:**

Synthesized Ag-NPs have a clear molluscicidal effect against various snail intermediate hosts of intestinal schistosome parasites and could potentially serve as next generation molluscicides.

## Author summary

Schistosomiasis is one of the major parasitic diseases with socio-economic and health importance in the developing world. Control of snail intermediate hosts of human schistosomes is an essential part of the program on fighting schistosomiasis. This study evaluated the effect of silver nanoparticles (Ag-NPs) on the snail intermediate hosts of *Schistosoma mansoni*, i.e. *Biomphalaria alexandrina*, *B*. *glabrata*, and the intermediate host of *S*. *japonicum*, i.e. *Oncomelania hupensis*. The results revealed that LC_50_ values of Ag-NPs against *B*. *alexandrina*, *B*. *glabrata* and *O*. *hupensis* were 7.91, 16.55 and 46.5 ppm, respectively, after 24 hrs of exposure. The results also revealed that *O*. *hupensis* snails tolerate better the toxic effect of Ag-NPs than *B*. *alexandrina* and *B*. *glabrata* snails. We also demonstrated that the freshwater crustacean *Daphnia magna* tolerate even higher concentration of Ag-NPs compared to the three species of snail intermediate hosts of schistosomes. Experiments on exposure of *B*. *alexandrina* to the sub-lethal concentration (LC_25_) of Ag-NPs obviously altered the antioxidant enzymes in the snail’s hemolymph. From the aforementioned results, Ag-NPs exhibited a promising molluscicidal activity against the snail intermediate hosts of human schistosomes, could eventually be used in the national schistosomiasis control program.

## Introduction

Schistosomiasis is a water-borne infectious disease transmitted by freshwater snails. Typically, people become infected when larval forms of the *Schistosoma* parasite–released by the snails–penetrate the skin during contact with infested water. Two forms of the disease are distributed in 78 countries of the world (mainly in tropical and subtropical areas): the urogenital schistosomiasis caused by *Schistosoma haematobium* infection, and the intestinal form of the disease caused mainly by *S*. *mansoni*, *S*. *japonicum*, *S*. *intercalatum*, *S*. *guineensis* and *S*. *mekongi* [1]. The three main species of blood fluke responsible of most of the human schistosomiasis cases are *S*. *mansoni*, *S*. *haematobium* and *S*. *japonicum*. With the progress of the national schistosomiasis control programs and the disease prevalence becoming lower in some areas, protection from schistosome infection is of more importance than treatment of human cases only. Therefore, there is an increasing need for effective control approaches in the national schistosomiasis control programs [2]. Two intestinal species that infect humans, *S*. *mansoni* and *S*. *japonicum*, are transmitted by *Biomphalaria* and *Oncomelania* snail genera, respectively. *Biomphalaria alexandrina*, one of the snail intermediate host of *S*. *mansoni*, is widely distributed in Egypt, other African and southern American countries [3], *B*. *glabrata*, also an intermediate host of *S*. *mansoni*, is distributed in many countries of South America and some of African countries [4], and *O*. *hupensis*, intermediate host of *S*. *japonicum* is distributed in China, Japan, Philippines and Indonesian island of Sulawesi [5].

Egypt is the first country in the world to initiate the national schistosomiasis control program. Since 1922, this control program has been on the schedule for the Ministry of Health and Population in Egypt [6] and various control strategies have already been implemented. Transmission control strategy followed the World Health Organization (WHO) recommendations: controlling the schistosomiasis transmission by reducing its snail intermediate hosts, along with anti-schistosomal medication of infected cases. As one of the important components for the national schistosomiasis control program, different molluscicide approaches have been tried, at different stages of control program. For example, in 1922, a national attack was launched against snails, together with the treatment of some seriously infected persons in Egypt. In 1926, the first planned control program was adopted at Dakhla oasis by using molluscicides, or copper sulfate, in all irrigation canals [7]. In 1961, molluscicides were applied in transmission sites, areas of land rehabilitation and in areas where seasonal irrigation was switched to perennial irrigation. The molluscicides, including copper sulfate, sodium pentachorophenate and niclosamide, were implemented in the snail ridden fields [7]. It should also be noted that the application of molluscicides is a procedure that is not only costly in application and necessary to be supported by great funds, but also has to be repeated for years to prevent snail from getting back into water systems again [8]. Besides, the application of molluscicides is toxic for the environment. Therefore, more cost-effective approaches were explored, including lining of canals with concrete to protect against the formation of snail colonies [9]. Plant molluscicides have also been applied in recent years in Egypt in the schistosomiasis control program [10]. In addition, biological control of snail has been implemented in the national schistosomiasis control programs: fishes (*Tilapia* spp.), ducks and aquatic insects have been used as natural predators or competitor of snail vectors [2].

On the other hand, nanotechnology is used to modify materials at the nano-scale (<100 nm) to create narrative properties. Changes in the physicochemical and structural properties of materials that resulted from the reduction in particle size can lead to new and sometimes unexpected biological effects [2]. The synthesis of metallic nanoparticles has become lately an active field of research, and more importantly in nanotechnology. Much attention has been paid to metallic nanoparticles because of their extraordinary physical and chemical properties, which broadly differ from their bulk properties [11]. Moreover, several studies analyzed the antibacterial mechanisms of action of silver (Ag) nano powder (Ag-NPs) on which numerous factors, such as NPs diameter, shape, surface changes, etc., has a great influence on the efficacy of antibiotics. Furthermore, nanoparticles are extensively used in the manufacture of textiles, for antimicrobial-property materials, as well as to control clothes odor, in food packaging, paints, cosmetics and biomedical products, mainly where antimicrobial properties are coveted [12]. Despite the silver antimicrobial properties, it is essential to investigate the potential toxic effects of Ag-NPs against different organisms including non-target species.

Previous studies have shown that molluscicides exhibit a broadly oxidative stress effect on snails. It is well known that oxidative stress occurs in organisms when the generation of oxygen radicals rate become superior to their decomposition rate [13]. Some essential antioxidants are presented during oxidative stress, but not all. Those antioxidants are: total antioxidants capacity (TAC), glutathione (GSH), nitric oxide (NO) and catalase (CAT), where TAC is the marker that evaluates the antioxidant status of the biological system and is very useful in estimating the response of the organism against the free radical production [13]. Reduced GSH is the first line of non-enzymatic antioxidant of the defense system that attacks the oxidative stress and gets depleted in the oxidative stress models [14]. CAT is the enzyme that changes superoxide into water and molecular oxygen and prevents the creation of hydroxyl radical and other toxic reactive oxygen species (ROS) [15]. It is also worth mentioning that most of ecotoxicological tests on hazardous materials using invertebrates had been conducted using crustaceans and not mollusks. Regarding the ecotoxicological tests conducted on manufactured nanomaterials, most tests have been done using the same testing organism species, such as a planktonic crustacean *Daphnia magna* [16].

The present study aimed to evaluate molluscicidal effects of Ag-NPs on *B*. *alexandrina*, *B*. *glabrata*, and *O*. *hupensis*, the snail intermediate hosts of either *S*. *mansoni* or *S*. *japonicum* blood flukes. At the same time, we investigated the potential molluscicidial mechanism(s) ongoing and we also assessed potential toxic effect of these Ag-NPs on other aquatic non-target organism, such as *Daphnia magna*.

## Materials and methods

### Ethics statement

Ethical clearance of this pilot had been granted approval by the Ethics Committee of the National Institute of Parasitic Diseases, Chinese Center for Disease Control and Prevention in Shanghai, China.

### Silver nanoparticles used

Ag-NPs (20 nm diameter) were purchased from Abvigen company, Newark, USA. In the 1—ethyl 3—methyl tetrafluoroborate, the electrochemical sacrificial anode method is used to prepare spherical silver nanoparticles (SNP) directly from the metal silver, in ultrasounds atmosphere. The structure, composition and morphology of the sample are characterized by the X-ray diffraction (XRD) and the transmission electron microscopy (TEM).

### Snail species used and rearing conditions

*B*. *alexandrina* and *B*. *glabrata* snails were obtained from Egyptian laboratory stock at Malacology Department, Theodor Bilharz Research Institute based in Cairo of Egypt, and *O*. *hupensis* snails from Chinese laboratory stock at National Institute of Parasitic Diseases, Chinese Center for Diseases Control and Prevention based in Shanghai of China, respectively. All snails were maintained at 25 ± 1°C, pH 7.4 and 12 hrs light / 12 hrs darkness photoperiod.

*B*. *alexandrina* and *B*. *glabrata* snails were maintained, as stock cultures, in a well-prepared snail room, under suitable environmental conditions, in glass aquaria containing dechlorinated tap water in a density of 10 snails / L. The snails were fed on fresh lettuce leaves, supplemented with tetramine (fish food) and chalk, after careful selection on both size and age basis.

*O*. *hupensis* snails were maintained in a breeding system consisting essentially of a mud-based aqua-terrarium for adult breeding snails and an aquarium for juvenile snails, respectively. The water in the aquarium connected with the terrarium through a pumping tube and a drain pipe. Newly hatched snails and egg masses are flushed into the aquarium by flooding the aquaterrarium once a fortnight. Normally the water level of the terrarium is kept just below the surface of the mud. When young snails reach a size of more than 3 mm in shell height they are moved on the mud into the aqua-terrarium, in order to meet all the needs of amphibious *O*. *hupensis* snails. Optimum area per snail was found to be 40 cm^2^/snail for breeding adults and 10 cm^2^/snail for growing juveniles. The optimum light intensity for breeding was produced by one 20 W 60 cm long fluorescent tube at 15 cm above the aquaterrarium. Both adult and juvenile snails are fed a modified Standen alginate food including dehydroacetic acid as an antiseptic [17].

### *D*. *magna* rearing conditions

*D*. *magna* were isolated from Lake Manzala (northeastern Egypt) and maintained in the laboratory at a constant temperature of 24±2°C, with a light/dark cycle of 12/12 hrs. *D*. *magna* were fed with the microalga, or *Scenedesmus obliquus*, three times a week. The food suspension was prepared by adding 14 x l0^8^ coenobia per liter of the freshwater synthetic medium for rearing. The synthetic medium is characterized by pH 7.9, conductivity 260 μmhos, alkalinity 34 mg/L as CaCO_3_ and total hardness 90 mg/L as CaCO_3_ [18]. Algae were grown in the culturing medium [19].

### Molluscicidal tests

A serial dilution of Ag-NPs was prepared in dH_2_O [20]. For each Ag-NPs concentration, 10 adult snails were used as targeted experimental snails. Each of the snail used measured 6–8 mm in diameter for *B*. *alexandrina* and *B*. *glabrata* or 6–8 mm in shell length for *O*. *hupensis*. All the experiments were conducted in triplicates. Each snail was subjected to static exposure to each of the concentration of Ag-NPs for 24, 48, 72hrs and 7 days, respectively, in plastic aquaria containing 1L volume of the test solution. Metal wires with narrow openings were used as barriers to prevent the snails from crawling out of the test solution. Snails were then allowed to recover for the next 24 hrs after the end of the exposure period. The results were documented on the next day of recovery, in order perform observations in a stabilized status. Control snails were maintained under the same experimental conditions in dechlorinated water [21]. The 100 μg/ml concentration of Ag-NPs was chosen empirically as a starting concentration. At this concentration, the lethal effect of Ag-NPs on the snails is 100%. Snails were considered dead if they did not move at all and showed a discolored body retracted into the shell for *B*. *alexandrina* and *B*. *glabrata* or if they did not move when punctured by a needle for *O*. *hupensis* [22]. Percentage of snail mortality was recorded and the LC_50_ and LC_90_ values of Ag-NPs were determined based on the results of these experiments.

In addition, *Daphnia magna*, the non-targeted organism, were exposed to the same dilution series of Ag-NPs, in order to calculate the LC_50_ and LC_90_ and compare these values to the ones obtained for the targeted snails, i.e. *B*. *alexandrina*, *B*. *glabrata* and *O*. *hupensis*.

### Antioxidant enzyme assays

About 1.5 ml of hemolymph was collected from each snail of *B*. *alexandrina* using the techniques according to Michelson [23], in order to measure the levels of antioxidant enzymes, such as TAC, GSH, CAT and nitric oxide synthase (NOS). An average of 100μL of hemolymph was used for each of assays. Antioxidant enzyme assays were calculated by colorimetric method in accordance with the instructions provided by Biodiagnostic company, and the kits purchased from Biodiagnostic, Dokki, Giza Governorate in Egypt (website: www.bio-diagnostic.com).

TAC level corresponds to the total antioxidative capacity performed by the reaction of antioxidants in the sample with a defined amount of exogenously provided hydrogen peroxide (H_2_O_2_). The antioxidants in the sample eliminated a certain amount of the provided hydrogen peroxide. The residual H_2_O_2_ quantity is determined colorimetrically by an enzymatic reaction which involves the conversion of 3,5,dichloro –2– hydroxy benzensulphonate to a colored product [24] (S1 File).

GSH reduced was measured based on the reduction of 5,5,dithiobis (2—nitrobenzoic acid) (DTNB) with glutathione to produce a yellow compound. The reduced chromogen absorbance, which is directly proportional to GSH concentration, can be measured at 405 nm [24] (S2 File).

CAT enzyme catalyzes the detoxification of H_2_O_2_ produced after the dismutation of O_2_ by superoxide dismutase (SOD) enzyme. It was assayed according to Lubinksy and Bewley (1979) using 10 mM H_2_O_2_. The decrease in absorbance was read at 230 nm and the activity of the enzyme was calculated using an extinction coefficient of 25 for the enzyme. A glycerophosphate dehydrogenase activity was assayed using d-glyceraldehyde 3-phosphate as substrate (10 mM) and nicotinamide adenine dinucleotide (NAD) (15 mM) in 0.1 M phosphate buffer, pH 7.0 [25]. The increase in absorbance was measured at 340 nm (S3 File).

NO enzyme is synthesized in biological system by the enzyme NOS. This NO is scavenged rapidly (t1/2 = 4 seconds) and acts in a paracrine fashion to a transducer of cellular signals. Thus, one of the indexes of NO production is the NO_2_. The endogenous nitrite concentration was measured in the hemolymph as an indicator of nitric oxide production using Biodiagnostic Nitrite Assay Kit (Griess Reagent). Addition of Griess Reagents to the hemolymph converts nitrite into a deep purple azo compound. Spectrophotometric measurement of this azo chromophore accurately determines NO_2_ concentration in snail hemolymph [26] (S4 File).

### Statistical analysis

Data were analyzed using Statistical Program for Social Science (SPSS) version 20.0 to calculate the LC_50_ and LC_90_ by probit analysis. Analytical statistics included one-way analysis of variance (F) (ANOVA), Chi-square test of significance, and post-hoc test (Dunnett method, the controls group is reference) were conducted. The confidence interval was set to 95% and the margin of error accepted was set to 5%. Probability (p-value) was calculated based on following criteria: p-value < 0.05 was considered significant.

## Results

### Characters of silver nanoparticles

**Fig 1:**
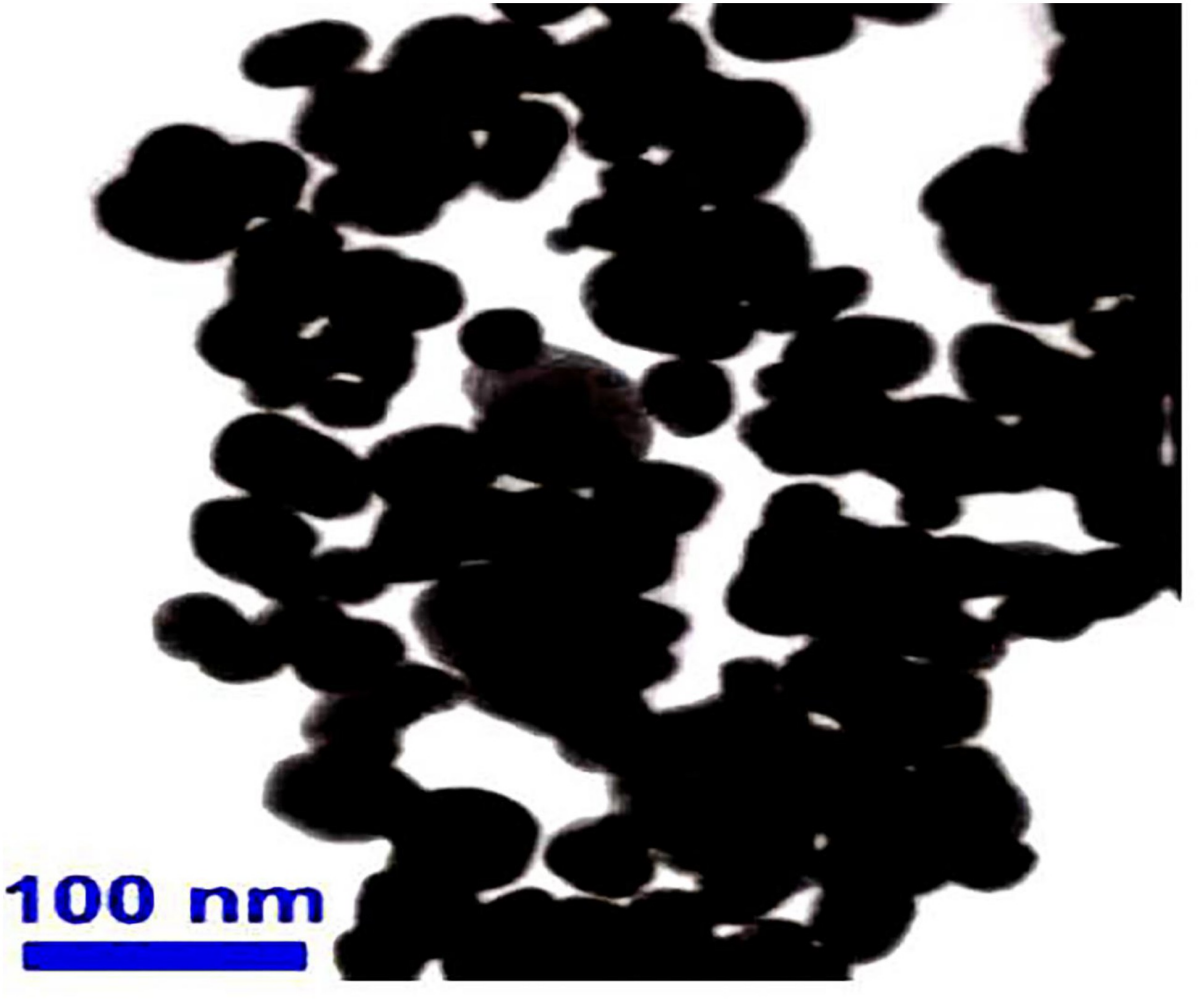
The morphology of silver nano powder illustrated by transmission electron microscope (TEM) (*Silver nano powder*: Type nano powder = diameter 20nm; Format powder = content 99.9%; Specific surface area = 10–20 m2/g; Density = 0.25–0.6 g/cm3; Storage = Stored at refrigerator).

The TEM images showed that Ag-NPs were mostly spherical in shape forming chain like aggregates with average particle size diameters 20nm. Elemental analysis of SNP performed using X-ray fluorescence revealed high purity of these synthesized particles; constituted at 99.99% by Ag (silver), with a specific surface area of 10–20 m^2^/g and a density of 0.25–0.6 g/cm^3^ (Fig 1).

### Molluscicidal test

**Fig 2:**
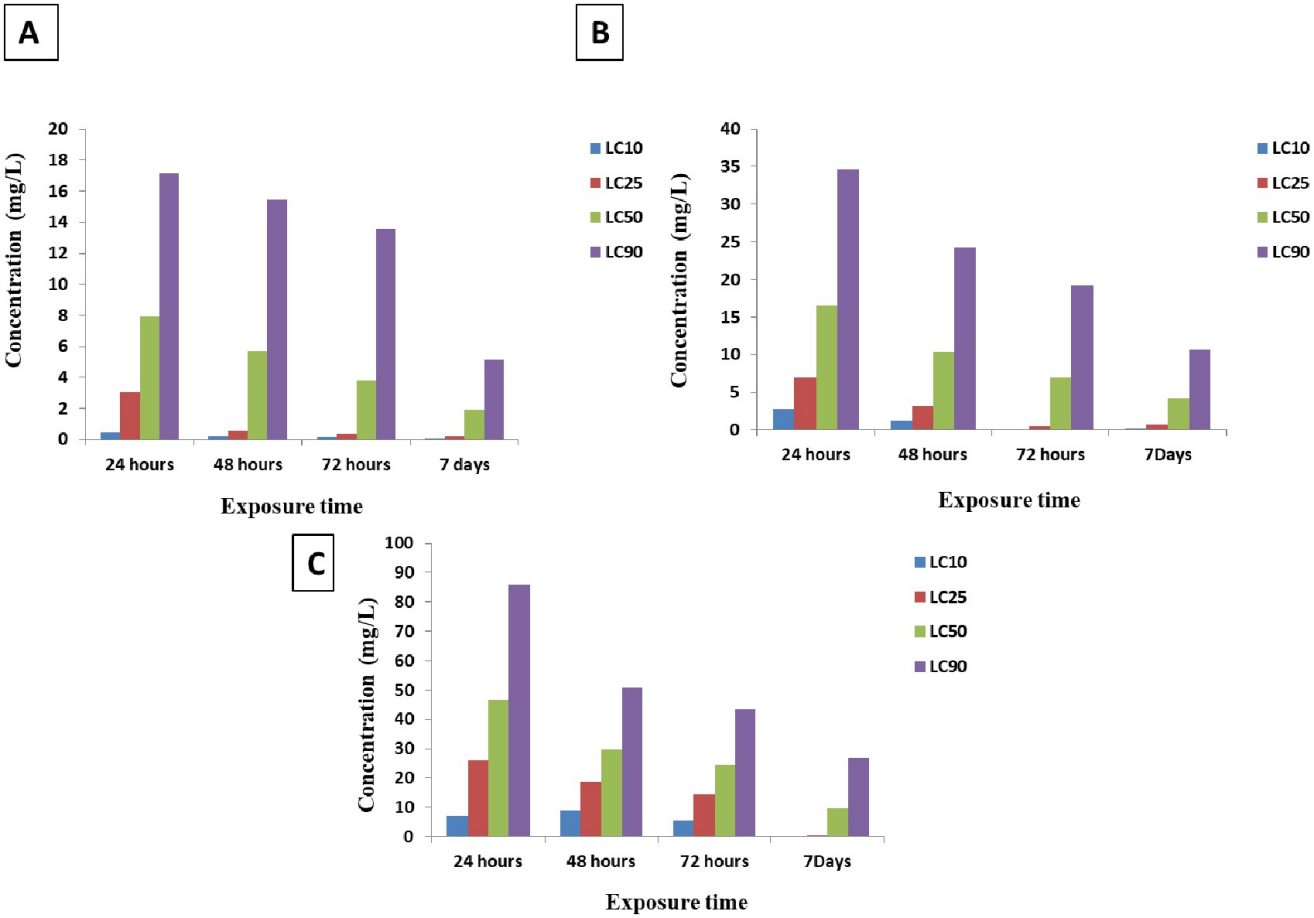
Differentiation of molluscicidal efficacy of Ag-NPs against A: *Biomphalaria alexandrina*, B: *Biomphalaria glabrata* and C: *Oncomelania hupensis* snail at different time point (24, 48, 72hrs and 7 days).

Table 1 shows Ag-NPs have toxic effect against the experimental snails (e.g. *B*. *alexandrina*, *B*. *glabrata* and *O*. *hupensis*). The values of LC_50_ after 24, 48, 72 hrs and 7 days for *B*. *alexandrina* snails were 7.91, 5.69, 3.83 and 1.91 ppm, respectively. Similarly, the values for *B*. *glabrata* were 16.55, 10.44, 6.91 and 4.13 ppm, respectively, and for *O*. *hupensis* were 46.5, 29.85, 24.49 and 9.62 ppm, respectively. This means that *B*. *alexandrina* snails are more susceptible to the toxic effect of Ag-NPs in comparison with *B*. *glabrata* and *O*. *hupensis* snails, which indicated that the order of snails susceptibility to the toxic effect of Ag-NPs was in the order of *B*. *alexandrinea > B*. *glabrata >O*. *hupensis*. While focusing on the variation of exposure effect of Ag-NPs on the three species of snails, we have found that the molluscicidal effect of Ag-NPs on *B*. *alexandrina* has more than double effects than their effect on *B*. *glabrata* (2.09 and 2.16 times more for 24hrs and 7 days exposure, respectively), and there was more than a quadrat effect when compared to Ag-NPs effect on *O*. *hupensis* (5.87 and 5.03 times more for 24hrs and 7 days exposure, respectively). But double molluscicidal effects were showed on *B*. *glabrata* (2.8 and 2.32 times more for 24hrs and 7 days exposure, respectively) when compared to *O*. *hupensis* (Fig 2 and S1–S6 Tables).

**Table 1:** Molluscicidal efficacy of the silver nanoparticles (Ag-NPs) against snail species.

Exposure time	snails	LC_10_ (95% CL) ppm	LC_25_ (95% CL) ppm	LC_50_ (95% CL) ppm	LC_90_ (95% CL) ppm
**24 hours**	***B*. *a***	**0.42(0.17–3.24)**	**3.04(1.05–5.43)**	**7.91(5.52–10.44)**	**17.16(13.79–24.19)**
	***B*. *g***	**2.8(1.4–7.6)**	**7.02(0.35–10.9**	**16.55(12.8–20.8)**	**34.64(28.3–47.6)**
	***O*.*h***	**7.20(3.7–17.2)**	**25.83(15.3–34.4)**	**46.52(37.6–60.1)**	**85.84(69.3–108.1)**
**48 hours**	***B*. *a***	**0.22(0.02–2.76)**	**0.569(.34–3.39)**	**5.698(2.73–8.24)**	**15.44(12.15–22.08)**
	***B*. *g***	**1.26(.98–4.6)**	**3.16(0.22–8.04)**	**10.44(7.02–13.4)**	**24.25(20.2–31.1)**
	***O*.*h***	**8.96(2.3–15.1)**	**18.86(11.4–23.5)**	**29.85(25.1–39.1)**	**50.74(40.8–78.4)**
**72 hours**	***B*. *a***	**0.15(.011–1.5)**	**0.383(0.03–3.1)**	**3.83(0.326–6.36)**	**13.54(10.3–20.6)**
	***B*. *g***	**0.17(0.09–2.3)**	**0.437(0.12–3.88)**	**6.91(3.35–9.7)**	**19.21(15.7–25.2)**
	***O*.*h***	**5.37(1.6–11.6)**	**14.43(5.9–18.8)**	**24.49(20.2–30.2)**	**43.62(35.8–63.6)**
**7 days**	***B*. *a***	**0.073(0.003–0.1)**	**0.183(0.02–1.37)**	**1.91(0.33–3.1)**	**5.1(3.7–10.8)**
	***B*. *g***	**0.27(0.02–1.4)**	**0.685(0.11–4.5)**	**4.13(1.9–6.1)**	**10.65(8.2–16.1)**
	***O*.*h***	**0.21(0.02–3.5)**	**0.50(.02–5.5)**	**9.62(4.2–14.1)**	**26.96(20.9–40.5)**

B. a: *Biomphalaria alexandrina*, B. g: *Biomphalaria glabrata* and O.h: *Oncomelaniahupensis*

Also, there is no mortality detected to *D*. *magna* (as a control group) which has been exposed to more than double and half concentration (50 ppm) of Ag-NPs (for ¼ hr, ½ hr, ¾ hr, 1hr, 2hrs and 3hrs, respectively) (S7 Table). As comparison, *B*. *alexandrina* snails LC_90_ is 18 ppm Ag-NPs.

### Antioxidant enzyme assays

In the antioxidant enzyme assays, *B*. *alexandrina* snail that exposed to sub-lethal concentration (LC_25_) of Ag-NPs for three consecutive days. Exposed snail hemolymph showed (i) a strong significant decrease in CAT activity (3.86± 0.01, 3.82±0.13 and 0.54± 0.08, respectively) when compared to control (i.e. unexposed snail hemolymph) group (24.77± 0.15); (ii) a strong significant decrease in GSH activity on the first day (6.43± 0.46), then a still significant decrease on the two other tested days (144.84±1.16 and 139.84± 1.49, respectively) when compared to control group (149.14± 1.96); (iii) a significant decrease in TAC activity on the first day (175.07± 2.19), followed by a stronger significant decrease on the other two days (68.98± 0.74 and 66.93± 2.02, respectively) when compared to control group (187± 1.45). On the other hand, there was a strong significant decrease in NO activity on the first two days (0.14± 0.01, 0.13±0.02, respectively), followed by a less strong but still significant decrease in NO on the third day (0.32± 0.01) when compare to control group (0.35± 0.02, Table 2) (S8 and S9 Tables).

**Table 2:** Antioxidant effects of *Biomphalaria alexandrina* snails after exposure to LC_25_ silver nanoparticles (Ag-NPs) for 24, 48 and 72 hrs.

Antioxidant	Control	24 hrs	48 hrs	72 hrs
**Catalase assay (U/ L)**	24.77± 0.15	**3.86± 0.01	**3.82±.13	**0.54± 0.08
**Glutathione (GSH), (mg/dl)**	149.14± 1.96	**6.43 ± 0.46	*144.84±1.16	*139.84± 1.49
**Total antioxidant capacity (mM/L)**	187.82± 1.45	*175.07± 2.19	**68.98± 0.74	**66.93± 2.02
**Nitric oxide assay (mM/L)**	0.35± 0.02	**0.14± 0.01	**0.13±0.02	*0.32± 0.01

* P< 0.05,

** P< 0.01 and

Data expressed as Mean± **SD**

## Discussion

Antimicrobial properties of ionic silver are known for a long time. The unique properties and behaviors of nanoparticles, as well as their potential interaction(s) and impact(s) on organisms, are particularly interesting in a context of environmental complexity [27]. In this study, Ag-NPs were selected to be tested as a promising molluscicides against three species of snail intermediate hosts of two human schistosome parasites, e.g. *S*. *mansoni* and *S*. *japonicum*. Our study showed that Ag-NPs had good molluscicidal effects with dose-dependent variations in lethal concentration. In our experiments, *B*. *alexandrina* was the most sensitive to the Ag-NPs molluscicidal effect compared to the other snail species tested. It was followed by *B*. *glabrata* and *O*. *hupensis*. Interestingly, *O*. *hupensis* was the less sensitive tested species to Ag-NPs exposure. The molluscicidal effect of Ag-NPs is in accordance with previous reports on the antimicrobial activity of Ag-NPs [28,29]. We suspected that Ag-NPs may generate free radicals which could damage/disturb cell lipidic membranes and eventually impair membrane functions [30,31].

Previous studies have indicated that the LC_50_ values of niclosamide for both *B*. *glabrata* and *B*. *straminea* were 0.06 mg/L [32]. Although niclosamide is more efficient against snails and has been recommended by WHO guidelines [33], it is also linked to many logistics problems associated with its application and its cost but also with its toxicity to non-target organisms including fishes. For example, niclosamide, being one of the causes of colon cancer, increased cell death when used as a therapeutic drug through hydrogen peroxide production [34], then it is not inconceivable that using it, directly or indirectly, for snail control. Therefore, it is urgent to find new lines of molluscicide, such as Ag-NPs, which exhibit strong molluscicidal effects combined with low toxicity to non-target organisms.

Our study also illustrated that three factors need to be considered to improve the molluscicidal effect on snails, including size, exposure time, and concentration of SNP. First, the size of SNP is an important factor which affect the surface of snail’s cell wall membrane. We used the Ag-NPs that was 20 nm in diameter, identified by TEM, in this study. This kind of Ag-NPs was considered to have the potential ability to adhere and be absorbed by the surface of targeted agents, eventually inducing snail mortality. Our finding was similar to previous report, that the surface properties of nanoparticles are directly linked to their aggregation behavior and that toxicity of Ag-NPs are due to the free silver ions coming from the nanoparticles [35]. Second, our study highlighted that the longer the snails’ exposure to SNP, the higher the molluscicidal activity of SNP to snails. A similar result was also observed by Yang et al. [36], who showed that all *Oncomelania* snails exposed to SNP were dead after 144 hrs [2]. Our study revealed that Ag-NPs molluscicidal activity is concentration-dependent (Fig 2). However, we have noticed important variations in the susceptibility of the different snail species tested to Ag-NPs, with LC_50_ ranged from double and quadrat values depending of the species. We hypothesized that this existing variations may be attributed to different binding abilities of cell membrane from the snail tegument in the three different snail species tested [37]. Finally, a cost-effectiveness study has to be performed before Ag-NPs could be applied in the field as a new line of molluscicide.

Microcrustacean *D*. *magna*is, one of the most widespread and sensitive species used for chemical and nanoparticle toxicity screening [38–40]. *Daphnia* spp. reproduce parthenogenetically, facilitating their maintenance and usage as a key toxicological model and indicator species for determining water quality. The present study highlighted that no mortality was recorded after *D*. *magna* had been exposed to more than double and half concentration of Ag-NPs for 3 hrs continuously, which usually cause 100% mortality to *B*. *alexandrina* snails. This result indicates that Ag-NPs are not toxic to non-target animals if used as molluscicide (requiring relatively low concentration of SNP). Our finding is in accordance with previous reports [41] showing a survival rate of 100% when *D*. *magna* are exposed to uncoated Ag-NPs, during their first generation. Furthermore, Hu et al. [42] also found that the bioaccumulation of Ag-NPs in *Daphnia* spp. and therefore, toxicity, was lower in the surface water, possibly as a result of the heavier aggregation compared to what is observed in culture M4 medium.

Several studies have already investigated the potential mechanism(s) of toxicity induced by Ag-NPs and found that their cytotoxicity is mainly mediated by the induction of ROS. In the present study, the antioxidant balance was evaluated by determining the levels of TAC, GSH, NO and CAT in snail hemolymph. We highlighted that all antioxidant enzymes activity decreases in the hemolymph of *B*. *alexandrina* when exposed to sub-lethal concentration of Ag-NPs. This result is in accordance to the existing literature, as a report by Ellis et al. that Ag-NPs considerably inhibited the enzymatic activity of CAT and SOD [41]. This enzymatic inhibition is induced by the interaction between Ag-NPs and the antioxidant enzymes, forming Ag-NP–protein complexes and potentially modifying the secondary structure of the enzymes, impairing their intrinsic activity. CAT is constituted of four identical subunits, which work together to perform its biological function in organisms. In order to reach the active center of CAT, H_2_O_2_ needs to diffuse inside a narrow channel made of 14 amino acids. The decrease in the activity of CAT could also be illustrated by the interaction of Ag^+^ ions released by Ag-NPs, and by amino acid residues within this narrow channel [43], impeding the approach of H_2_O_2_ to the active center and decreasing the soluble CAT activity [36].

## Conclusion

**Fig 3:**
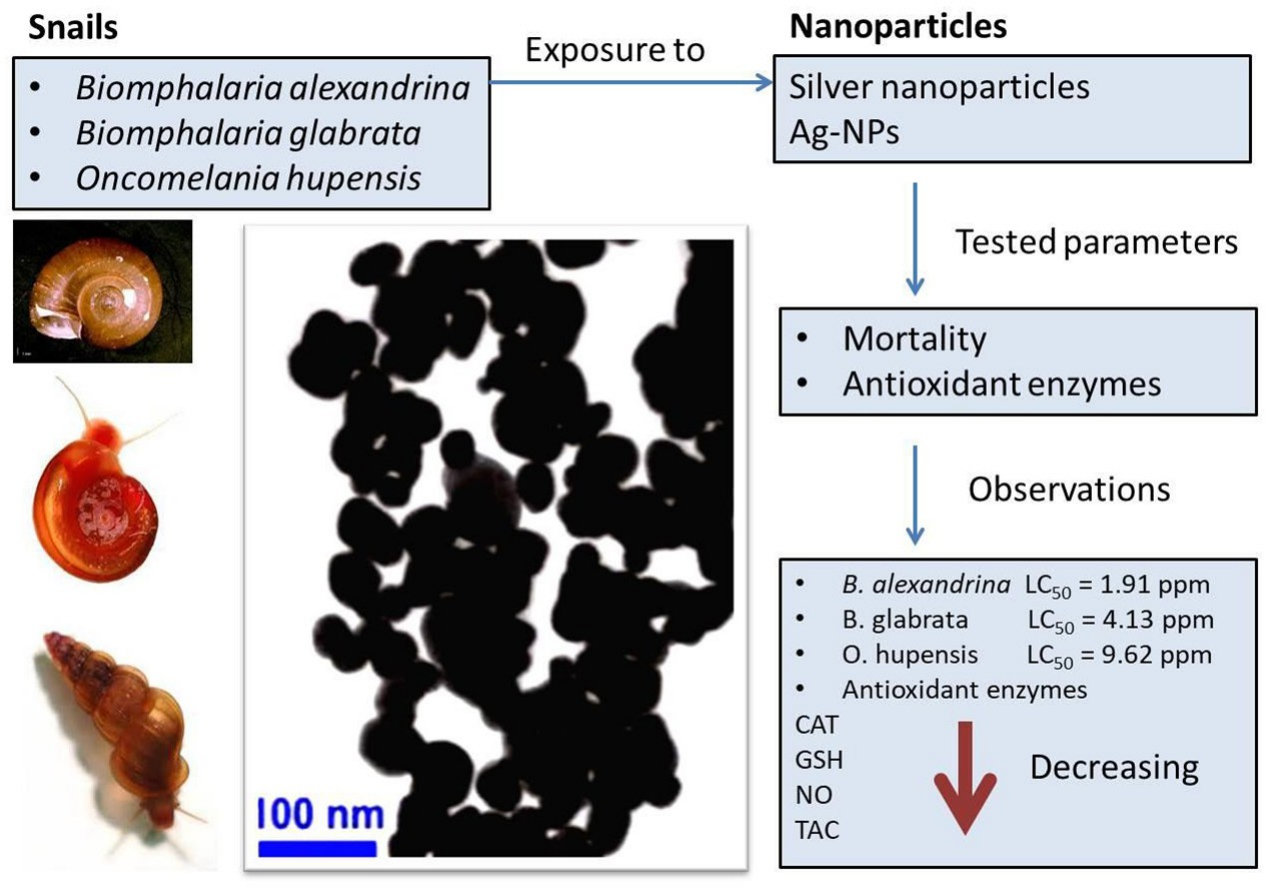
Graphical abstract.

The present study demonstrated the potential applications of Ag-NPs as molluscicide in the national schistosomiasis control programs. Indeed, Ag-NPs are efficient in killing three species of snail, i.e. *B*. *alexandrina*, *B*. *glabrata* and *O*. *hupensis*, which are considered major snail intermediate hosts of the human schistosomes parasites, including *S*. *mansoni* and *S*. *japonicum* (Fig 3). The lethal concentration of Ag-NPs varied between the three species of snails tested, while no toxicity to the other aquatic non-target organisms has been observed. Therefore, Ag-NPs may become a new and promising molluscicide for extensive application in the field to control schistosomiasis. The exposure to Ag-NPs induced a decrease in antioxidant enzyme activity, e.g. TAC, GSH, NO and CAT, in snail hemolymph has raised a hypothesize that this decrease in antioxidant enzyme activity could be due to the formation of complexes between Ag-NPs and enzymes, indicating that these interactions are strong enough to promote conformational changes in the enzymatic structure and leading to a strong decrease in the snail’s antioxidant capacity.

## Supporting information

S1 Table Mortality of *B*. *alexandrina* after exposure to silver nanoparticles (Ag-NPs). (DOCX)

S2 Table Mortality of *B*. *glabrata* after exposure to silver nanoparticles (Ag-NPs). (DOCX)

S3 Table Mortality of *O*. *hupensis* after exposure to silver nanoparticles (Ag-NPs). (DOCX)

S4 Table Acute and chronic molluscicidal activity of the compounds against adult *B*. *alexandrina* snail. (DOCX)

S5 Table Acute and chronic molluscicidal activity of the testes compounds against adult *B*. *glabrata* snail. (DOCX)

S6 Table Acute and chronic molluscicidal activity of the compounds against adult *O*. *hupensis* snail. (DOCX)

S7 Table Molluscicidal activity of silver nanoparticles (Ag-NPs) against adult *Daphnia magna*. (DOCX)

S8 Table Summary of antioxidant parameters of *B*. *alexandrina* in each assay after exposure to silver nanoparticles (Ag-NPs). (DOCX)

S9 Table Detail results of antioxidant parameters of *B*. *alexandrina* in each assay after exposure to silver nanoparticles (Ag-NPs), including section 1 of S9 Table (Results of Catalase assay, in mU/ L); section 2 of S9 Table (Results of Glutathione reduced (GSH) assay, in mg/dl); section 3 of S9 Table (Results of total antioxidant capacity assay, in mM/L); section 4 of S9 Table (Results of Nitric oxide assay, in μmol / L). (DOCX)

S1 File Methodology of Total Antioxidant Capacity (TAC) Assay. (PDF)

S2 File Methodology of Glutathione (GSH) Assay. (PDF)

S3 File Methodology of Catalase (CAT) Assay. (PDF)

S4 File Methodology of Nitric Oxide (NO) Assay. (PDF)
