# Supplementary material for: Molluscicidal and antioxidant activities of silver nanoparticles on the multi-species of snail intermediate hosts of schistosomiasis
Source: PLoS Negl Trop Dis. 2022 Oct 10;16(10):e0010667. doi: 10.1371/journal.pntd.0010667 (PMC9550036; doi:10.1371/journal.pntd.0010667)
Supplement: S1 Table — (DOCX) [file pntd.0010667.s001.docx]

**S1 Table.**  **Mortality of *B. alexandrina***

| S | control | one day | Two days | Three days | 7 days |  |
| --- | --- | --- | --- | --- | --- | --- |
| 0.5 | 0/10 | 0 | 1 | 2 | 2 |  |
| 1 | 0/10 | 1 | 2 | 3 | 5 |  |
| 3 | 0/10 | 2 | 4 | 5 | 6 |  |
| 5 | 0/10 | 5 | 7 | 8 | 9 |  |
| 10 | 0/10 | 6 | 8 | 8 | 10 |  |
| 15 | 0/10 | 7 | 8 | 8 | 10 |  |
| 18 | 0/10 | 9 | 9 | 10 | 10 |  |
| 21 | 0/10 | 9 | 10 | 10 | 10 |  |
| 24 | 0/10 | 10 | 10 | 10 | 10 |  |
