# Supplementary material for: Molluscicidal and antioxidant activities of silver nanoparticles on the multi-species of snail intermediate hosts of schistosomiasis
Source: PLoS Negl Trop Dis. 2022 Oct 10;16(10):e0010667. doi: 10.1371/journal.pntd.0010667 (PMC9550036; doi:10.1371/journal.pntd.0010667)
Supplement: S3 Table — (DOCX) [file pntd.0010667.s003.docx]

**S3 Table. Mortality of *O. hupensis***

| Conce. | control | 24 | 48 | 72 | 7 DAY |  |
| --- | --- | --- | --- | --- | --- | --- |
| 5 | 0/10 | 0 | 0 | 1 | 6 |  |
| 10 | 0/10 | 0 | 2 | 2 | 6 |  |
| 15 | 0/10 | 1 | 2 | 2 | 7 |  |
| 18 | 0/10 | 2 | 3 | 4 | 7 |  |
| 21 | 0/10 | 3 | 3 | 4 | 7 |  |
| 24 | 0/10 | 3 | 3 | 5 | 8 |  |
| 27 | 0/10 | 3 | 4 | 5 | 8 |  |
| 30 | 0/10 | 4 | 4 | 6 | 9 |  |
| 40 | 0/10 | 5 | 8 | 9 | 10 |  |
| 80 | 0/10 | 7 | 10 | 10 | 10 |  |
| 100 | 0/10 | 10 | 10 | 10 | 10 |  |
