# Supplementary material for: Molluscicidal and antioxidant activities of silver nanoparticles on the multi-species of snail intermediate hosts of schistosomiasis
Source: PLoS Negl Trop Dis. 2022 Oct 10;16(10):e0010667. doi: 10.1371/journal.pntd.0010667 (PMC9550036; doi:10.1371/journal.pntd.0010667)

| **compound** | **LC_10_  (95% CL)**  **Ppm** | **LC_25_ (95% CL)**  **Ppm** | **LC_50_ (95% CL)**  **Ppm** | **LC_90_ (95% CL)**  **Ppm** | **slope** |
| --- | --- | --- | --- | --- | --- |
| **Silver NP exposure 24 houre** | **0.42 (0.17- 3.24)** | **3.04(1.05- 5.43)** | **7.91 (5.52- 10.44)** | **17.16 (13.79-24.19)** | **9.95** |
| **Silver NP exposure 48 houre** | **0.22 (.023- 2.76)** | **.569(.34- 3.39)** | **5.698(2.73-8.24)** | **15.44(12.15-22.08)** |  |
| **Silver NP exposure 72 houre** | **0.15 (.011-1.5)** | **0.383(0.03-3.1)** | **3.83(.326-6.36)** | **13.54(10.3-20.6)** |  |
| **Silver NP exposure 7Day** | **0.073(0.003-0.1)** | **.183(0.02-1.37)** | **1.91(.33-3.1)** | **5.1(3.7-10.8)** |  |

**S4 Table. Acute and chronic molluscicidal activity of the compounds against adult *B. alexandrina* snail**


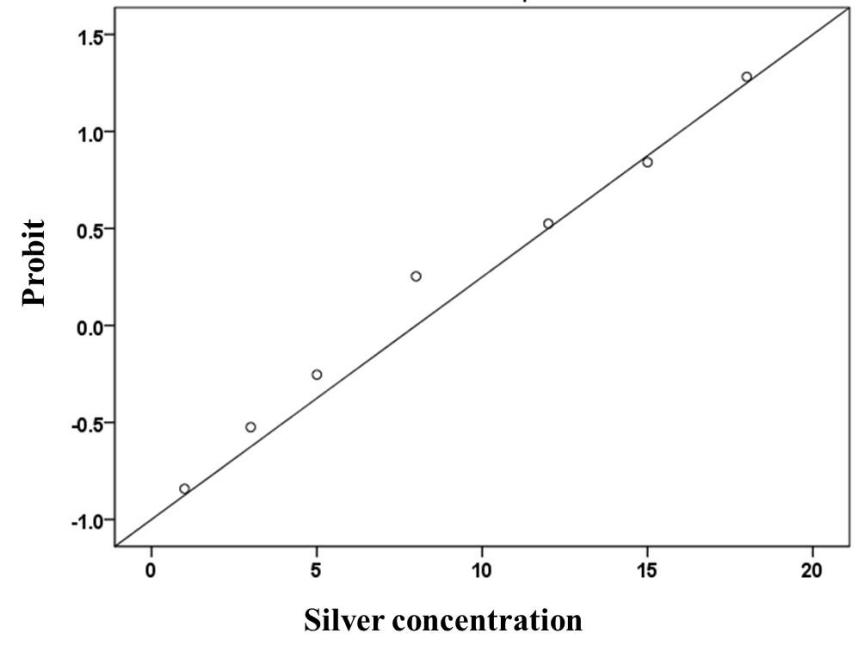

Supplement: S4 Table — (DOCX) [file pntd.0010667.s004.docx]
