# Supplementary material for: Molluscicidal and antioxidant activities of silver nanoparticles on the multi-species of snail intermediate hosts of schistosomiasis
Source: PLoS Negl Trop Dis. 2022 Oct 10;16(10):e0010667. doi: 10.1371/journal.pntd.0010667 (PMC9550036; doi:10.1371/journal.pntd.0010667)
Supplement: S5 Table — (DOCX) [file pntd.0010667.s005.docx]

**S5 Table. Acute and chronic molluscicidal activity of the testes compounds against adult *B. glabrata* snail**

| **compound** | **LC_10_  (95% CL)**  **Ppm** | **LC_25_ (95% CL)**  **Ppm** | **LC_50_ (95% CL)**  **Ppm** | **LC_90_ (95% CL)**  **Ppm** | **slope** |
| --- | --- | --- | --- | --- | --- |
| **Silver NP exposure 24 houre** | 2.8(1.4-7.6) | 7.02(.35-10.9 | 16.55(12.8-20.8) | 34.64(28.3-47.6) |  |
| **Silver NP exposure 48 houre** | 1.26(.98-4.6) | 3.16(.22-8.04) | 10.44(7.02-13.4) | 24.25(20.2-31.1) |  |
| **Silver NP exposure 72 houre** | 0.17(0.09-2.3) | .437(0.12-3.88) | 6.91(3.35-9.7) | 19.21(15.7-25.2) |  |
| **Silver NP exposure 7Day** | 0.27(0.02-1.4) | 0.685(0.11-4.5) | 4.13(1.9-6.1) | 10.65(8.2-16.1) |  |
