# Supplementary material for: Molluscicidal and antioxidant activities of silver nanoparticles on the multi-species of snail intermediate hosts of schistosomiasis
Source: PLoS Negl Trop Dis. 2022 Oct 10;16(10):e0010667. doi: 10.1371/journal.pntd.0010667 (PMC9550036; doi:10.1371/journal.pntd.0010667)
Supplement: S6 Table — (DOCX) [file pntd.0010667.s006.docx]

**S6 Table. Acute and chronic molluscicidal activity of the compounds against adult *O. hupensis*  snail**

| **compound** | **LC_10_  (95% CL)**  **Ppm** | **LC_25_ (95% CL)**  **Ppm** | **LC_50_ (95% CL)**  **Ppm** | **LC_90_ (95% CL)**  **Ppm** | **slope** |
| --- | --- | --- | --- | --- | --- |
| **Silver NP exposure 24 houre** | 7.20(3.7-17.2) | 25.83(15.3-34.4) | 46.52(37.6-60.1) | 85.84(69.3-108.1) |  |
| **Silver NP exposure 48 houre** | 8.96(2.3-15.1) | 18.86(11.4-23.5) | 29.85(25.1-39.1) | 50.74(40.8-78.4) |  |
| **Silver NP exposure 72 houre** | 5.37(1.6-11.6) | 14.43(5.9-18.8) | 24.49(20.2-30.2) | 43.62(35.8-63.6) |  |
| **Silver NP exposure 7Day** | .21(0.02-3.5) | .50(.02-5.5) | 9.62(4.2-14.1) | 26.96(20.9-40.5) |  |
