# Supplementary material for: Molluscicidal and antioxidant activities of silver nanoparticles on the multi-species of snail intermediate hosts of schistosomiasis
Source: PLoS Negl Trop Dis. 2022 Oct 10;16(10):e0010667. doi: 10.1371/journal.pntd.0010667 (PMC9550036; doi:10.1371/journal.pntd.0010667)
Supplement: S7 Table — (DOCX) [file pntd.0010667.s007.docx]

**S7 Table. Molluscicidal activity of the Silver NP against adult *Daphnia magna***

| Concentration | 15min | 30 min | 45 min | 1h | 2h | 3h |
| --- | --- | --- | --- | --- | --- | --- |
| 10 ppm | 0% | 0% | 0% | 0% | 0% | 0% |
| 20 ppm | 0% | 0% | 0% | 0% | 0% | 0% |
| 30 ppm | 0% | 0% | 0% | 0% | 0% | 0% |
| 40 ppm | 0% | 0% | 0% | 0% | 0% | 0% |
| 50 ppm | 0% | 0% | 0% | 0% | 0% | 0% |
