# Supplementary material for: Molluscicidal and antioxidant activities of silver nanoparticles on the multi-species of snail intermediate hosts of schistosomiasis
Source: PLoS Negl Trop Dis. 2022 Oct 10;16(10):e0010667. doi: 10.1371/journal.pntd.0010667 (PMC9550036; doi:10.1371/journal.pntd.0010667)
Supplement: S8 Table — (DOCX) [file pntd.0010667.s008.docx]

**S8 Table. Antioxidant parameters of *B.* *alexandrina***

| Anti-oxidant parameter | 24hrs exposure | 48hrs exposure | 72hrs exposure | Control |
| --- | --- | --- | --- | --- |
| Catalase assay ( mU/ L) | (3.86, 3,88, 3,85) | (3.9, 3.82, 3.3) | (0.42, 0.53, 0.56) | (24.8, 24.6, 24.9) |
| Glutathione reduced (GSH), ( mg/dl) | (5.99, 6.4, 6.9) | (145.98, 143.66, 144.87) | (141.3, 139.88, 138.33) | (147.31, 148.89, 151.21) |
| Total antioxidant capacity ( mM/L) | (176.92, 175.64, 172.66) | 68.18, 69.65, 69. 11) | 69.23, 66.12, 65.45) | (186.67, 187.33, 189.45) |
| Nitric oxide assay  ( mM/L) | (0.13, 0.15, 0.15) | (0.23, 0.11, 0.14) | (0.33, 0.32, 0.31) | (0.33, 0.35, 0.36) |
