# Supplementary material for: Molluscicidal and antioxidant activities of silver nanoparticles on the multi-species of snail intermediate hosts of schistosomiasis
Source: PLoS Negl Trop Dis. 2022 Oct 10;16(10):e0010667. doi: 10.1371/journal.pntd.0010667 (PMC9550036; doi:10.1371/journal.pntd.0010667)
Supplement: S9 Table — (DOCX) [file pntd.0010667.s009.docx]

**S9 Table. Anti-oxidant parameter**

**S9-1 Table.** **Catalase assay ( mU/ L)**

| 24h exposure | 48h exposure | 72h exposure | Control |
| --- | --- | --- | --- |
| **3.86** | **3.9** | **0.42** | **24.8** |
| **3.88** | **3.82** | **0.53** | **24.6** |
| **3.85** | **3.85** | **0.56** | **24.9** |

**One-way ANOVA: 24h exposure; 48h exposure; 72h exposure; Control_1**

| Tests of Normality | Shapiro-Wilk | | |
| --- | --- | --- | --- |
| Catalase assay ( U/L) | Statistic | df | P |
| 24h expo | 0.977 | 3 | 0.712 |
| 48h expo | 0.999 | 3 | 0.956 |
| 72h expo | 0.995 | 3 | 0.862 |
| Control | 0.964 | 3 | 0.637 |

| Test of Homogeneity of Variances | Levene Statistic | df1 | df2 | P |
| --- | --- | --- | --- | --- |
| Catalase assay ( U/L) |  |  |  |  |
| Based on Mean | 0.5 | 3 | 8 | 0.693 |
| Based on Median | 0.246 | 3 | 8 | 0.862 |
| Based on Median and with  adjusted df | 0.246 | 3 | 6.117 | 0.861 |
| Based on trimmed mean | 0.481 | 3 | 8 | 0.704 |

| Catalase assay ( U/L) | Sum of Squares | df | Mean Square | F | P |
| --- | --- | --- | --- | --- | --- |
| Between Groups | 1113.402 | 3 | 371.134 | 27172.714 | <0.001 |
| Within Groups | 0.109 | 8 | 0.014 |  |  |
| Total | 1113.511 | 11 |  |  |  |

| (I) group | (J) group | Mean Difference (I-J) | Std. Error | P | 95% Confidence Interval | |
| --- | --- | --- | --- | --- | --- | --- |
|  |  |  |  |  | Lower Bound | Upper Bound |
| 24h exposure | Control | -20.90333 | 0.09542 | <0.001 | -21.1781 | -20.6285 |
| 48h exposure | Control | -20.94333 | 0.09542 | <0.001 | -21.2181 | -20.6685 |
| 72h exposure | Control | -24.23 | 0.09542 | <0.001 | -24.5048 | -23.9552 |

Dunnett t (2-sided)

**S9-2 Table. Glutathione reduced (GSH), ( mg/dl)**

| Control | 24h exposure | 48h exposure | 72h exposure |
| --- | --- | --- | --- |
| **147.31** | **5.99** | **145.98** | **141.3** |
| **148.89** | **6.4** | **143.66** | **139.88** |
| **151.21** | **6.9** | **144.87** | **138.33** |

**One-way ANOVA: contrl; 24h exposure; 48h exposure; 72h exposure**

| Tests of Normality | Shapiro-Wilk | | |
| --- | --- | --- | --- |
| GSH ( U/L) | Statistic | df | P |
| 24h expo | 0.997 | 3 | 0.891 |
| 48h expo | 0.999 | 3 | 0.952 |
| 72h expo | 0.999 | 3 | 0.952 |
| Control | 0.988 | 3 | 0.792 |

| GSH ( U/L) | Levene Statistic | df1 | df2 | P |
| --- | --- | --- | --- | --- |
| Based on Mean | 1.107 | 3 | 8 | 0.401 |
| Based on Median | 0.79 | 3 | 8 | 0.533 |
| Based on Median and  with adjusted df | 0.79 | 3 | 5.211 | 0.548 |
| Based on trimmed mean | 1.087 | 3 | 8 | 0.408 |

| GSH ( U/L) | Sum of Squares | df | Mean Square | F | P |
| --- | --- | --- | --- | --- | --- |
| Between Groups | 43086.688 | 3 | 14362.229 | 7550.226 | <0.001 |
| Within Groups | 15.218 | 8 | 1.902 |  |  |
| Total | 43101.905 | 11 |  |  |  |

| (I) group | (J) group | Mean Difference (I-J) | Std. Error | P | 95% Confidence Interval | |
| --- | --- | --- | --- | --- | --- | --- |
|  |  |  |  |  | Lower Bound | Upper Bound |
| 24h exposure | Control | -142.70667 | 1.12612 | <0.001 | -145.9495 | -139.4638 |
| 48h exposure | Control | -4.3 | 1.12612 | 0.013 | -7.5428 | -1.0572 |
| 72h exposure | Control | -9.3 | 1.12612 | <0.001 | -12.5428 | -6.0572 |

**Dunnett t (2-sided)**

**S9-3 Table. Total antioxidant capacity ( mM/L)**

| Control | 24h exposure | 48h exposure | 72h exposure |
| --- | --- | --- | --- |
| **186.67** | **176.92** | **68.18** | **69.23** |
| **187.33** | **175.64** | **69.65** | **66.12** |
| **189.45** | **172.66** | **69.11** | **65.45** |

| Tests of Normality | Shapiro-Wilk | | |
| --- | --- | --- | --- |
| Total antioxidant capacity ( U/L) | Statistic | df | P |
| 24h expo | 0.95 | 3 | 0.568 |
| 48h expo | 0.977 | 3 | 0.71 |
| 72h expo | 0.878 | 3 | 0.319 |
| Control | 0.916 | 3 | 0.438 |

| Total antioxidant capacity ( U/L) | Levene Statistic | df1 | df2 | P |
| --- | --- | --- | --- | --- |
| Based on Mean | 1.54 | 3 | 8 | 0.277 |
| Based on Median | 0.322 | 3 | 8 | 0.81 |
| Based on Median and with  adjusted df | 0.322 | 3 | 5.851 | 0.81 |
| Based on trimmed mean | 1.395 | 3 | 8 | 0.313 |

| Total antioxidant capacity ( U/L) | Sum of Squares | df | Mean Square | F | P |
| --- | --- | --- | --- | --- | --- |
| Between Groups | 38888.677 | 3 | 12962.892 | 4505.463 | <0.001 |
| Within Groups | 23.017 | 8 | 2.877 |  |  |
| Total | 38911.695 | 11 |  |  |  |

| (I) group | (J) group | Mean Difference (I-J) | Std. Error | P | 95% Confidence Interval | |
| --- | --- | --- | --- | --- | --- | --- |
|  |  |  |  |  | Lower Bound | Upper Bound |
| 24h exposure | Control | -12.74333 | 1.38495 | <0.001 | -16.7315 | -8.7551 |
| 48h exposure | Control | -118.83667 | 1.38495 | <0.001 | -122.8249 | -114.8485 |
| 72h exposure | Control | -120.88333 | 1.38495 | <0.001 | -124.8715 | -116.8951 |

Dunnett t (2-sided)

**S9-4 Table. Nitric oxide assay**

| Control | 24h exposure | 48h exposure | 72h exposure |
| --- | --- | --- | --- |
| **0.33** | **0.13** | **0.13** | **0.33** |
| **0.35** | **0.15** | **0.11** | **0.32** |
| **0.36** | **0.15** | **0.14** | **0.31** |

| Tests of Normality | Shapiro-Wilk | | |
| --- | --- | --- | --- |
| Nitric oxide assay | Statistic | df | P |
| 24h expo | 0.923 | 3 | 0.463 |
| 48h expo | 0.964 | 3 | 0.637 |
| 72h expo | 1 | 3 | 1 |
| Control | 0.964 | 3 | 0.637 |

| Nitric oxide assay | Levene Statistic | df1 | df2 | P |
| --- | --- | --- | --- | --- |
| Based on Mean | 0.79 | 3 | 8 | 0.533 |
| Based on Median | 0.19 | 3 | 8 | 0.9 |
| Based on Median and  with adjusted df | 0.19 | 3 | 5.765 | 0.899 |
| Based on trimmed mean | 0.731 | 3 | 8 | 0.562 |

| Nitric oxide assay | Sum of Squares | df | Mean Square | F | P |
| --- | --- | --- | --- | --- | --- |
| Between Groups | 0.113 | 3 | 0.038 | 150.167 | <0.001 |
| Within Groups | 0.002 | 8 | 0 |  |  |
| Total | 0.115 | 11 |  |  |  |

| (I) group | (J) group | Mean Difference (I-J) | Std. Error | P | 95% Confidence Interval | |
| --- | --- | --- | --- | --- | --- | --- |
|  |  |  |  |  | Lower Bound | Upper Bound |
| 24h exposure | Control | -0.19 | 0.01291 | <0.001 | -0.2272 | -0.1528 |
| 48h exposure | Control | -0.22 | 0.01291 | <0.001 | -0.2572 | -0.1828 |
| 732h exposure | Control | -0.02667 | 0.01291 | 0.166 | -0.0638 | 0.0105 |

Dunnett t (2-sided)
