# Supplementary material for: Molluscicidal and antioxidant activities of silver nanoparticles on the multi-species of snail intermediate hosts of schistosomiasis
Source: PLoS Negl Trop Dis. 2022 Oct 10;16(10):e0010667. doi: 10.1371/journal.pntd.0010667 (PMC9550036; doi:10.1371/journal.pntd.0010667)
Supplement: S1 File — (PDF) [file pntd.0010667.s010.pdf]

Supplementary File S1.
Methodology of Total Antioxidant Capacity (TAC) Assay

TOTAL ANTIOXIDANT CAPACITY

Colorimetric Method **50 Tests**

For Research Only

INTRODUCTION :

Reactive oxygen species (ROS) are produced as a consequence of normal aerobic metabolism. Unstable free radical species attack cellular components causing damage to lipids, proteins, and DNA which can initiate a chain of events resulting in the onset of a variety of diseases. Living organisms have developed complex antioxidant systems to counteract ROS and to reduce their damage. These antioxidant systems include enzymes such as superoxide dismutase, catalase,and glutathione peroxidase; macromolecules such as albumin, ceruloplasmin, and ferritin; and an array of small molecules, including ascorbic acid, α-tocopherol, β-carotene, reduced glutathione, uric acid, and bilirubin. The sum of endogenous and food-derived antioxidants represents the total antioxidant activity of the system. The cooperation among different antioxidants provides greater protection against attack by reactive oxygen or nitrogen species, than any single compound alone. Thus, the overall antioxidant capacity may provide more relevant biological information compared to that obtained by the measurement of individual components, as it considers the cumulative effect of all antioxidants present in plasma and body fluids.

PRINCIPLE :

The determination of the antioxidative capacity is performed by the reaction of antioxidants in the sample with a defined amount of exogenously provide hydrogen peroxide (H_2O_2) The antioxidants in the sample eliminate a certain amount of the provided hydrogen peroxide. The residual H_2O_2 is determined colorimetrically by an enzymatic reaction which envolves the conversion of 3,5,dichloro –2– hydroxy benzensulphonate to a colored product.

STABILITY :

Stable until the expiry date specified when stored at +4 to +8 °C

REAGENTS :

**1.**	Substrate (H_2O_2)<br>**(Dilute 1000 times before use)**
**2.**	Chromogen
**3.**	Enzyme – Buffer

PROCEDURE :

- **Dilute $R_1$1000 times immediately before use(10 µl R1 + 10 ml d. Water mix .) Discard after use .**
- **Working Reagent : mix equal volumes of R2 and R3 immediately before use .**
- **Dilute sample if necessary**

	**Blank ml**	**Sample ml**
**d. H_2O**	**0.02**	**-**
**Sample**	**-**	**0.02**
**R1 (substrate)**	**0.50**	**0.50**
**Mix well. Incubate 10 min at 37°C then add:**		
**Working reagent**	**0.5**	**0.5**
**Mix well . Incubate 5 min. at 37°C**		
**Read immediately the absorbances of blank (A_B) and sample (A_{SA}) against d. water at 505 (500 – 510 nm) . Linearity up to 2 mM / L .**		

CALCULATION :

Total Antioxidant concentration

$$\text{mM / L} = A_B - A_{SA} \times 3.33$$

REFERENCE VALUE:

Serum or Plasma	:	0.5 - 2 mM / L
Urine	:	0.2 – 3 mM / L
Saliva	:	0.3 – 1 mM / L

N.B .

To obtain reproducible results, antioxidant levels of the sample should fall within the values of the standard curve. When necessary, samples can be diluted with saline to bring antioxidants to this level. Multiply the result by the dilution factor .

SAMPLE PREPARATION

Plasma

1. Collect blood using an anticoagulant such as heparin or citrate. Do not use EDTA.
2. Centrifuge the blood at 3000 rpm for 10 min. at 4°C. pipette off the top yellow plasma layer without disturbing the white buffy layer. Store plasma on ice until assaying or freeze at -80°C. The plasma sample will be stable for at least one month.

Serum

1. Collect blood without using an anticoagulant Allow blood to clot for 30 min. at 25°C.
2. Centrifuge the blood at 3000 rpm for 15 min. at 4°C. pipette off the top yellow serum layer without disturbing the white buffy layer. Store serum on ice if not assaying the same day, freeze at -80°C. The sample will be stable for at least one month.

Urine .

Collect urine in a clear beaker or flask and store on ice. If not assaying the same day, freeze at -80°C.

Saliva

Collect saliva in a clear beaker or flask and store on ice. If not assaying the same day, freeze at -80°C.

Cell Lysate

1. Collect cells by centrifugation (i.e., 1000-2000 rpm for 10 min. at 4°C) . For adherent cells, do not harvest using proteolytic enzymes; rather use a rubber policeman.
2. The cell pellet can be homogenized or sonicated on ice in 1-2 ml of cold buffer (i.e., 5 mM potassium phosphate, pH 7.4. containing 0.9% sodium chloride and 0.1%glucose).
3. Centrifuge at 4000 rpm for 15 min. at 4°C.
4. Remove the supernatant for assay and store on ice if not assaying the same day, freeze at -80°C. The sample will be stable for at least one month.

REFERENCE :

Koracevic, D., Koracevic, G., et al. J . Clin . Pathol. 54, 356 – 361 (2001)
EL –Aaser, A.A. (unpublished)

BIODIAGNOSTIC
DIAGNOSTIC AND RESEARCH REAGENTS

TOTAL ANTIOXIDANT CAPACITY

Colorimetric Method

+4 to +8ºC **50 Tests**

CAT. NO. TA 25 13

FOR RESEARCH USE ONLY

REAGENTS

**R1**	Substrate	**2**	**ml**
**R2**	Chromogen	**15**	**ml**
**R3**	Enzyme - Buffer	**15**	**ml**

CONTACTS

Tele: 02-33385184
Mobil: 0109 – 349 20 77
Fax : 02-33385184 (102)
e.maile : info@bio-diagnostic.com
Website: www.bio-diagnostic.com
Adress: 29 Tahreer St., Dokki, Giza, Egypt
