# Supplementary material for: Molluscicidal and antioxidant activities of silver nanoparticles on the multi-species of snail intermediate hosts of schistosomiasis
Source: PLoS Negl Trop Dis. 2022 Oct 10;16(10):e0010667. doi: 10.1371/journal.pntd.0010667 (PMC9550036; doi:10.1371/journal.pntd.0010667)
Supplement: S2 File — (PDF) [file pntd.0010667.s011.pdf]

Supplementary File S2.
Methodology of Glutathione (GSH) Assay

SAMPLE PREPARATION

Tissue Homogenate

1. Prior to dissection, perfuse tissue with a PBS (phosphate buffered saline) solution, pH 7.4 . containing 0.16 mg / ml heparin to remove any red blood cells and clots.
. Homogenize the tissue in 5 – 10 ml cold buffer (i , e , 50 mM potassium phosphate, pH7.5.1 mM EDTA) per gram tissue,using tissue homogenizer.
3. Centrifuge at 4,000 rpm for 15 minutes at 4°C .
4. Remove the supernatant for assay and store on ice. If not assaying on the same day , freeze the sample at - 80°C. The sample will be stable for at least one month.

Cell Lysate

1. Collect cells by centrifugation (i ,e, 1,000 – 2,000 rpm for 10 minutes at 4 °C). For adherent cells, do not harvest using proteolytic enzymes; rather use a rubber policeman .
2. Homogenize cell pellet in cold buffer (i, e, 50 mM potassium phosphate, pH 7.5. 2 mM EDTA) .
3. Centrifuge at 4,000 rpm for15 minutesat 4 °C .
4. Remove the supernatant for assay and store on ice. If not assaying on the same day , freeze the sample at - 80°C. The sample will be stable for at least one month.

Wholeblood and Erythrocyte Lysate

1. Collect blood using an anticoagulant such as heparin, citrate , or EDTA .
2. whole blood can be used for assay.
3. Lyse the erythrocytes (red blood cells) in 4 times its volume of ice – cold distilled water .
4. Centrifuge at 4,000 rpm for 15 minutes at 4 °C .
5. Collect the supernatant (erythrocyte lysate) for assaying and store on ice. If not assaying on the same day , freeze at - 80°C. The sample will be stable for at least one month.

REFERENCE :

Beutler E. , Duron O. , Kelly MB.
J. Lab Clin. Med. (1963) , 61 , 882

BIODIAGNOSTIC
DIAGNOSTIC AND RESEARCH REAGENTS

GLUTATHIONE REDUCED

Colorimetric Method

(R1+R3) +4 to +8˚C — 50 Tests
(R2) +15 to +25˚C

CAT. No. **GR 25 11**

FOR RESEARCH USE ONLY

REAGENTS

R1	TCA	25 ml
R2	Buffer	50 ml
R3	DTNB	5 ml

CONTACTS

Tele: 02-33385184
Mobil: 0109 – 349 20 77
Fax : 02-33385184 (102)
e.maile : info@bio-diagnostic.com
Website: www.bio-diagnostic.com
Adress: 29 Tahreer St., Dokki, Giza, Egypt

GLUTATHIONE REDUCED (GSH)

Colorimetric Method **50 Tests**
For research only

PRINCIPLE :

The method based on the reduction of 5,5` dithiobis (2 - nitrobenzoic acid) (DTNB) with glutathione (GSH) to produce a yellow compound . The reduced chromogen directly proportional to GSH concentration and its absorbance can be measured at 405 nm.

SAMPLE :

Fresh heparinized blood and appropriate concentration of fresh tissue homogenate

REAGENTS :

1.	Trichloroacetic acid (TCA)	500 mmol / L
2.	Buffer	100 mmol / L
3.	DTNB	1.0 mmol / L

STABILITY:

Stable until the expiry date specified when stored at +4 to +8 °C for R1+ R3 and at +15 to +25 °C for R2

PROCEDURE:

	Blood ml	Tissue ml	Blank ml
Sample	0.1	0.5	-
Dis. Water	0.5	-	0.5
Reagent 1	0.5	0.5	0.5
Mix well , allow to stand for 5 min. at R . T. Centrifuge at 3000 rpm for 15 min. then take the following aliquots :			
Supernate	0.5	0.5	0.5
Reagent 2	1.0	1.0	1.0
Reagent 3	0.1	0.1	0.1

Mix well. Measure the absorbance after 5-10 min. at 405 nm of sample (A_{Sample}) against the blank. Linearity up to 120 mg/dL (4 mmol/L)

CALCULATION :

Glutathione (GSH) concentration

In blood $= A_{Sample} \times 66.66$ mg/dL

$= A_{Sample} \times 2.22$ mmol/L

In Tissue $= \frac{A_{Sample} \times 66.66}{\text{g. tissue used}}$ mg / g. tissue

$= \frac{A_{Sample} \times 2.22}{\text{g. tissue used}}$ mmol / g. tissue
