# Supplementary material for: Molluscicidal and antioxidant activities of silver nanoparticles on the multi-species of snail intermediate hosts of schistosomiasis
Source: PLoS Negl Trop Dis. 2022 Oct 10;16(10):e0010667. doi: 10.1371/journal.pntd.0010667 (PMC9550036; doi:10.1371/journal.pntd.0010667)
Supplement: S3 File — (PDF) [file pntd.0010667.s012.pdf]

Supplementary File S3.
Methodology of Catalase (CAT) Assay

CATALASE ASSAY

Colorimetric Method **25 Tests**
For Research Use Only

INTRODUCTION :

Catalase is antioxidant enzyme that is present in most aerobic cells. It serves as one of the body's defense systems against H_2O_2, a strong oxidant that can cause intracellular damage. It is found in high concentration in erythrocytes and liver while lower concentrations are found in skeletal muscle, brain and heart. Measurement of catalase activity can be useful as a research tool for certain diseases such as acute pancreatitis and some liver diseases where values are elevated. Each unit of catalase decomposes 1µM of H_2O_2 per minute at 25°C and pH 7.0. Catalase assay adds to the family of Antioxidant Biomarkers and provides another useful tool for oxidative stress investigations.

PRINCIPLE :

Catalase reacts with a known quantity of H_2O_2. The reaction is stopped after exactly one minute with catalase inhibitor .

$$2\,H_2O_2 \xrightarrow{\text{Catalase}} 2\,H_2O + O_2$$

In the presence of peroxidase (HRP), remaining H_2O_2 reacts with 3,5-Dichloro -2-hydroxybenzene sulfonic acid (DHBS) and 4-aminophenazone (AAP) to form a chromophore with a color intensity inversely proportional to the amount of catalase in the original sample.

$$2\,H_2O_2 + \text{DHBS} + \text{AAP} \xrightarrow{\text{HRP}} \text{Quinoneimine Dye} + 4H_2O$$

REAGENTS :

**1.**	Buffer :<br>Phosphate buffer, pH 7.0<br>Detergent	100 mM / L
**2.**	H_2O_2 (substrate and standard)<br>(Dilute 1000 times before use)	500 mM / L
**3.**	Chromogen - Inhibitor	
**4.**	Enzyme :<br>Peroxidase<br>4 – Aminoantipyrine<br>Preservative	> 2000 / L<br>2 mM / L

PROCEDURE :

Dilute R2 1000 times immediately before use . (10 uL + 10 ml d. Water) . Discard after use .
Dilute Sample if necessary.

	Sample Blank (ml)	Sample (ml)	Standard Blank (ml)	Standard (ml)
**Sample**	0.05	0.05	-	-
**D. H_2O**	0.05	-	0.10	0.05
**R1**	0.50	0.50	0.50	0.50
**R2**	-	0.10	-	0.10
**Incubate exactly ONE min. at 25ºC then add :**				
**R3**	0.20	0.20	0.20	0.20
**R4**	0.50	0.50	0.50	0.50

Incubate 10 min. at 37ºC , read sample (A_{Sample}) against sample blank and standard ($A_{Standard}$) against Standard blank at 510 nm (500 – 520 nm). Color stable for one hour .

CALCULATION :

Catalase Activity :

In Plasma (U / L) =

$$\frac{A_{standard} - A_{Sample}}{A_{standard}} \times 1000$$

In Tissue (U / g) =

$$\frac{A_{standard} - A_{Sample}}{A_{standard}} \times \frac{1}{\text{gm tissue used per test}}$$

REFERENCE :

Aebi , H. (1984) Methods Enzymol 105, 121 – 126
Fossati, P., et.al . (1980) Clin. Chem. 26 , 227 - 231

SAMPLE PREPARATION

Tissue Homogenate

1. Prior to dissection, perfuse tissue with a PBS (phosphate buffered saline) solution, pH 7.4 . containing 0.16 mg / ml heparin to remove any red blood cells and clots.
2. Homogenize the tissue in 5 – 10 ml cold buffer (i , e , 50 mM potassium phosphate, pH 7.4. 1 mM EDTA and 1 mL/L Triton X-100) per gram tissue.
3. Centrifuge at 4,000 rpm for 15 minutes at 4 °C .
4. Remove the supernatant for assay and store on ice. If not assaying on the same day , freeze the sample at - 80°C. The sample will be stable for at least one month.

Plasma

1. Collect blood using an anticoagulant such as heparin, citrate , or EDTA .
2. Centrifuge at 4,000 rpm for 15 minutes at 4 °C .
3. Collect the plasma for assaying and store on ice. If not assaying on the same day, freeze at - 80°C. The sample will be stable for at least one month.

BIODIAGNOSTIC
DIAGNOSTIC AND RESEARCH REAGENTS

CATALASE ASSAY

Colorimetric Method

+4 to +8ºC **25 Tests**

CAT. No. CA 25 17

FOR RESEARCH USE ONLY

REAGENTS

**R1**	Buffer	**25 ml**
**R2**	H_2O_2	**2 ml**
**R3**	Chromogen-Inhibitor	**10 ml**
**R4**	Enzyme	**25 ml**

CONTACTS

Tele: 02-33385184
Mobil: 0109 – 349 20 77
Fax : 02-33385184 (102)
e.maile : info@bio-diagnostic.com
Website: www.bio-diagnostic.com
Adress: 29 Tahreer St., Dokki, Giza, Egypt
