# Supplementary material for: Molluscicidal and antioxidant activities of silver nanoparticles on the multi-species of snail intermediate hosts of schistosomiasis
Source: PLoS Negl Trop Dis. 2022 Oct 10;16(10):e0010667. doi: 10.1371/journal.pntd.0010667 (PMC9550036; doi:10.1371/journal.pntd.0010667)
Supplement: S4 File — (PDF) [file pntd.0010667.s013.pdf]

Supplementary File S4.
Methodology of Nitric Oxide (NO) Assay

NITRIC OXIDE ASSAY

Colorimetric Determination of Nitrite
For Research Only 25 Test

INTRODUCTION :

Nitric Oxide (NO) is synthesized in biological system by the enzyme Nitric Oxide Synthase (NOS) . NOS is a remarkably complex enzyme which acts on molecular oxygen, arginine, and NADPH to produce NO, citrulline, and NADP+ .

$$O_2 + \text{Arginine} + \text{NADPH} \xrightarrow{\text{NOS}} \text{. NO} + \text{Citrulline} + \text{NADP}$$

NO is produced in trace quantities by neurons, endothelial cells, platelets, and neutrophils in response to homeostatic stimuli. This NO is scavenged rapidly ($t_{1/2}$ = 4 seconds) and acts in a paracrine fashion to transduce cellular signals. NO is also produced by other cells (macrophages, fibroblasts, hepatocytes) in micromolar concentrations in response to inflammatory or mitogenic stimuli.
The final products of NO in vivo are nitrite (NO_2^-) and nitrate (NO_3^-) . The relative proportion of NO_2^- and NO_3^- produced from NO is variable . The exogenous source of NO_3 ingested in the diet should be considred and can not be ignored (none-NO origine) . Thus, one of the index of NO production is the NO_2 . The Biodiagnostic Nitrite Assay Kit provides an accurate and convenient method for measurement of endogenous nitrite concentration as indicatore of nitric oxide production in biological fluides . It depend on the addition of Griess Reagents which convert nitrite into a deep purple azo compound, photometric measurement of the absorbance due to this azo chromophore accurately determines NO_2^- concentration .

PRINCIPLE :

In acid medium and in the presence of nitrite the formed nitrous acid diazotise sulphanilamide and the product is coupled with N-(1–naphthyl) ethylenediamine. The resulting azo dye has a bright reddish – purple color which can be measured at 540 nm .

REFERENCE :

Montgomery , H. A . C and Dymock, J . F . (1961) Analyst, 86 , 414

SAMPLE :

urine, saliva, cytosol of tissue homogenate filtered or centrifuged .
Store at -20°C to -80 °C .

REAGENTS :

**1.**	Standard sodium nitrite	50 µmol/L
**2.**	Sulphanilamide	10 mmol/ L
**3.**	N – (1 – naphthyl) - ethylenediamine (NEDA)	1 mmol/ L

STABILITY :

The reagents are stable up to the expiry date specified when store at +4 to +8 °C .

PROCEDURE:

	Sample ml	Sample blank ml	Standard ml	Standard Blank ml
**Sample**	**0.1**	**0.1**	**-**	**-**
**R1**	**-**	**-**	**0.1**	**0.1**
**R2**	**1.0**	**1.0**	**1.0**	**1.0**
**Mix well, allow to stand for 5 min , then add :**				
**R3**	**0.1**	**-**	**0.1**	**-**

Mix well, allow to stand for 5 min. Read absorbance of sample (A_{sample}) against sample blank and of standard ($A_{standard}$) against standard blank at 540 nm (520 – 550 nm) Color stable for many hours . Linearity up to 200 µmol / L .

CALCULATION :

Nitrite in sample

$$\mu mol / L = \frac{A_{Sample}}{A_{Standard}} \times 50$$

Biodiagnostic

Sample Preparation

The kit has been validated in urine, culture media. No sample purification from these sources is necessary other than some special instructions as described below. Store samples at -20 °C or – 80 °C after collection.

1. Urine Sample

Urine can be used directly after dilution to the proper concentration .

2. Saliva.

Collect saliva in a clear beaker and store on ice . If not assayed in the same day freeze at -80 .

3. Tissue homogenates

1-Prior to dissection, perfuse tissue with a PBS (phosphate buffered saline) solution, pH 7.4. containing 0.16 mg / ml heparin to remove any red blood cells .

2.-Homogenize the tissue in 5 –10 ml cold buffer (i,e ,100 mM potassium phosphate, pH 7.0, containing 2 mM EDTA) per gram tissue.

3-. Centrifuge at 4,000 rpm for 15 minutes at 4 °C.

4- Remove the supernatant for assay and store on ice. If not assaying on the same day , freeze the sample at - 80°C. The sample will be stable for at least one month.

4. Culture Media

Some types of tissue culture media contain very high nitrate levels. These types of media should not be used for cell culture if the goal of an experiment is to measure small changes in nitrate levels. Cellular nitrite production can be quantitated by subtracting the level of nitrite present in the media (in the absence of cells) from the total nitrite level present during cell growth.The effect of media components on color development can be assessed by making a nitrite standard curve in the presence of a fixed volume of the culture media and comparing it to a nitrite standard curve made in buffer alone.

Interferences

Antioxidants will interfere with the color development reaction. Azide, ascorbic acid, dithiothreitol,and mercaptoethanol will interfere with color development when present at concentration as low as 100µM..Alkyl amines, most sugars, lipids, or amino acid (except those containing thiol groups) do not interfere.

Sensitivity

When using the maximum amount of sample for the nitrite assay (100µl), the detection limit is 2.5 µM.

NITRIC OXIDE ASSAY

Colorimetric Determination of Nitrite

+4 to +8ºC	25Test
CAT. No.	NO 25 33

FOR RESEARCH USE ONLY

REAGENTS

**R1**	Standard	**5**	**ml**
**R2**	Sulphanilamide	**50**	**ml**
**R3**	NEDA	**5**	**ml**

www.bio-diagnostic.com

29 Tahreer St., Dokki, Giza, Egypt
TeleFax : 3385184
e.mail : biodiagnostic _eka@lycos.com
info@bio-diagnostic.com
customerservice@bio-diagnostic.com

Biodiagnostic
